# A Single Perioperative Dose of Dexamethasone Is Not Associated With an Increased Risk of Infection in Patients With Hip or Knee Osteoarthritis Considered for Elective Primary Total Hip or Total Knee Arthroplasty

**DOI:** 10.1016/j.artd.2025.101761

**Published:** 2025-07-02

**Authors:** Samo Roškar, Vesna Levašič, Mateja Blas, Simon Kovač

**Affiliations:** a Valdoltra Orthopaedic Hospital, Ankaran, Slovenia; b Faculty of Medicine, University of Ljubljana, Ljubljana, Slovenia; c Valdoltra Orthopaedic Hospital, The National Arthroplasty Registry of Slovenia, Ankaran, Slovenia; d Faculty of Medicine, University of Maribor, Maribor, Slovenia

**Keywords:** Total hip arthroplasty, Total knee arthroplasty, Dexamethasone, Arthroplasty registry, Outcome

## Abstract

**Background:**

Perioperative dexamethasone administration has been associated with reduced postoperative nausea, pain, and enhanced recovery after total hip (THA) and knee arthroplasty (TKA). However, there is ongoing concern about the influence of glucocorticoids on periprosthetic joint infection (PJI) due to the high heterogeneity of available studies. The present study aimed to evaluate the effect of perioperative dexamethasone use on early and delayed PJI after THA and TKA in patients with primary osteoarthritis.

**Methods:**

A consecutive cohort of patients from a single teaching hospital were analyzed. For each procedure, the intravenous application of 0.15 mg/kg dexamethasone in a single dose perioperatively was recorded. Additionally, patient characteristics, joint affected and the revision surgery due to infection after primary surgery were collected from the hospital registry and medical records. All patients with revision surgery for PJI were subjected to the standard protocol of perioperative diagnostics.

**Results:**

Between 2017 and 2023, a total of 8905 procedures were included, 1318 with perioperative dexamethasone application and 7587 without. The patients who received perioperative dexamethasone (dexa group) were similar in age (effect size (ES) = 0.03), sex (ES = 0.002), body mass index (ES = 0.06) and American Society of Anaesthesiologists score (ES = 0.06) compared to patients who did not (non-dexa group). In total, 54 PJIs were observed, 6 of them were in dexamethasone and 48 in the non-dexamethasone group. Multivariate logistic regression showed that the use of dexamethasone was not associated with higher odds for PJI within the first 2 postoperative years (odds ratio 0.80 95% confidence interval 0.30-1.73; *P* = .603).

**Conclusions:**

Our study is the first single-dose, single-hospital study with standardized PJI diagnostic procedures at revision surgery, which shows that perioperative intravenous dexamethasone has no association with an increased risk of early or delayed PJI in THA and TKA in patients with primary osteoarthritis. The present data provide evidence in support of the perioperative administration of a single dose of dexamethasone.

## Introduction

Perioperative corticosteroid administration has been associated with multiple benefits associated with reduced postoperative nausea, pain, and enhanced recovery after total hip (THA) and knee arthroplasty (TKA) [1,2]. It decreases key inflammatory processes including cell proliferation and cytokine secretion, and it has been speculated to be associated with increased postoperative infection risk [3,4]. Dexamethasone is a long-acting high-potency glucocorticoid commonly used in postoperative pain management [1]. Perioperative administration has been associated with postoperative hyperglycaemia, which is known to be associated with poor wound healing which is one of the major risk factors for postoperative periprosthetic joint infection (PJI) [3,5,6]. Uncontrolled diabetes and increased body mass index (BMI) can potentiate potentially negative effects of dexamethasone role on early PJI [3]. Some studies report the increased PJI rate in the perioperative dexamethasone cohort [4], while some newer studies available report perioperative safety of dexamethasone use regarding PJI [2,7]. Several recently published systemic reviews and metanalyses evaluate the effect of dexamethasone on total joint arthroplasty [8,9] and found no significant difference in PJI occurrence between the dexamethasone and the non-dexamethasone group [9,10]. However, all metanalyses emphasize the existence of significant heterogeneity in studies available regarding dosing regimens, inclusion criteria for patients receiving perioperative corticosteroids and further research required. A recent registry study by Heckmann et al. is the first significantly powered study which shows no association of intraoperative dexamethasone use with increased risk of PJI within 90 days postoperatively [11].

The present study aimed to evaluate the effect of preoperative dexamethasone on PJI incidence after THA and TKA in patients with primary osteoarthritis within the first 2 postoperative years operated with the same cohort of surgeons in the same surgical environment.

## Material and methods

In the period from January 1, 2017, to January 1, 2023, a total of 6170 THAs and 3984 TKAs were implanted in a single teaching hospital. Out of these, a cohort of 8905 consecutive surgery procedures in patients that met the inclusion criteria of primary osteoarthritis was considered for elective THA or TKA procedures. To analyze acute and delayed infections all the patients completed minimal 2-year postoperative follow-up period at the time of analysis [12].

Patient demographics, sex, age, BMI, joint affected (hip/knee) and the date of revision surgery due to infection after primary surgery were collected from the patients charts, and missing data were retrieved from the hospital arthroplasty registry [13]. The hospital arthroplasty registry was additionally cross-checked with the National arthroplasty registry. The completeness of the National registry for the in-house primary procedures is 100%, while the completeness for the revision procedures is 94%, what means that 6% of the arthroplasties primary operated in the Valdoltra Orthopaedic Hospital were revised elsewhere. Considering BMI, all patients managed in our hospital in reported period of time were included with no lower or upper border of BMI for a patient to be excluded. In addition, American Society of Anaesthesiologists (ASA) score and the presence of diabetes and the preoperative application (15-30 minutes before the start of the surgery) of a single dose of 0.15 mg/kg dexamethasone was collected from the hospital medical database. The use of dexamethasone was primarily influenced by the attending anesthesiologist. Due to different approaches to anesthesia, some anesthesiologists tended to avoid using dexamethasone, while other (mostly younger) anesthesiologists were more inclined to administer it. Elective arthroplasty was performed only in patients with well-controlled diabetes (hemoglobin A1C < 7%).

According to hospital revision surgery protocol, at each revision surgery, joint fluid was examined for cell count and differential, at least 5 tissue samples for microbiology and histopathology were obtained and sonication of the prosthesis (only polyethylene liner in TKA) was performed in all the cases [14]. Definite diagnosis of PJI was based on the European Bone and Joint Infection Society (EBJIS) criteria [12]. For the patients having the revision of prosthetic joint before 2021, retrospective analysis was performed using data from the hospital database. This analysis was based on documented preoperative and intraoperative diagnostic findings relevant to the EBJIS criteria. The criteria for PJI were either sinus tract present or positive cell exam from synovial fluid aspirate or at least 2 positive intraoperative microbiological cultures with the same microorganism or positive sonication of the explanted material with >50 colony-forming unit/mL or positive histopathological exam of intraoperative tissue samples. Thus, all PJI cases in the study have fulfilled the EBJIS PJI-confirmed category [12].

All the surgeries were performed by or under the supervision of 11 high-volume surgeons (more than 150 joint arthroplasties per year). Patients were operated in 3 operating theaters equipped with vertical laminar flow. Perioperative antibiotic prophylaxis consisted of 2 g of intravenous cefazoline preoperatively, followed by 3 additional dosages of 1 g of cefazoline. Vancomycin was used in patients with a history of cephalosporin allergy. All patients without contraindications received a single preoperative dose of 15 mg/kg body weight tranexamic acid followed by 2 g of tranexamic acid injected in the joint cavity before the end of the surgery. Dalteparin was used after the surgery for 35 days as thromboembolic prophylaxis except in patients with preoperative anticoagulation therapy, where the preoperative therapy was prescribed after the surgery. In all cases of cemented fixation of the implant, premixed gentamicin loaded bone cement was used. Patients were mobilized on the day of the surgery, and weight-bearing as tolerated was allowed at patients' discretion.

### Data analysis

Categorical data were summarized as frequencies (%) and numeric data as medians (interquartile range). The variables age, sex, BMI, ASA, diabetes, type of procedure and administration of dexamethasone were included in univariate analysis and thereafter the multivariate logistic regression with the same factors was performed. The use of single 0.15 mg/kg dose of dexamethasone (dexa) and the non-dexa groups were compared with a chi-square test or Mann–Whitney test. The magnitude of the difference between groups, ie, effect size (ES) was calculated using Cohen’s *d* and Cramer’s V coefficient. The association between infection and the use of dexamethasone was estimated using multiple logistic regression. Analysis was adjusted for sex, BMI, ASA score, and operated joint. All analyses were performed with the software R version 4.4.1 (Foundation for Statistical Computing, Vienna, Austria).

## Results

A total of 1318 patients received perioperative dexamethasone (dexa group) and 7587 patients did not (non-dexa group). The patients who received perioperative dexamethasone (dexa group) were similar in age (ES = 0.03), sex (ES = 0.002), BMI (ES = 0.06), and ASA score (ES = 0.06) compared to patients who did not (non-dexa group). Additionally, the percentage of patients with diabetes mellitus was similar in both groups (ES = 0.03) as was the percentage of operated hips and knees (ES = 0.02) (Table 1). Among patients who received dexamethasone, 793 had THA and 525 TKA and in the non-dexa group, there were 4389 THA and 3198 TKA. For our cohort, 97.3% of knee arthroplasties were cemented and the remaining 2.7% were cementless. While in THA, most are done cementless 97.8%, the remaining 2.2% were hybrid arthroplasties with cemented femoral component. In all cases with cemented fixation, the antibiotic loaded cement was used.

**Table 1:** Patient characteristics in dexa and non-dexa groups.

Characteristc	Dexa n = 1318	Non-dexa n = 7587	*P* value	ES (95% CI)
Age (y)	69 (62.4-75.2)	69.1 (62.7-75.4)	.477	0.03 (−0.03 to 0.08)
Sex			.881	0.002 (0.00-0.023)
male	584 (44%)	3345 (44%)		
female	734 (56%)	4242 (56%)		
BMI (kg/m^2^)	29.6 (26.5-33.1)	29.9 (26.7-33.6)	.033	0.06 (0.00-0.12)
ASA				
I-II	891 (68%)	4529 (60%)	<.001	0.06 (0.04-0.08)
III-IV	427 (32%)	3058 (40%)		
Diabetes				
no	1209 (92%)	6764 (89%)	.005	0.03 (0.01-0.05)
yes	109 (8%)	823 (11%)		
Joint				
hip	793 (60%)	4389 (58%)	.115	0.02 (0.00-0.04)
knee	525 (40%)	3198 (42%)		

Univariate analysis revealed no significant association between the PJIs and the use of dexamethasone (odds ratio (OR) 0.72, 95% confidence interval (CI) 0.27-1.55; *P* = .446). Among the other factors examined, the significant were BMI (*P* < .001), ASA score (*P* = .003), and sex (*P* = .006) when PJI within 2 years since primary surgery is defined as the end point (Table 2). When revision due to any cause is defined as the end-point only BMI and ASA were statically significant risk factors. There were 6 of 1318 (0.5%) PJIs in the dexa group and 48 of /7587 (0.5%) PJIs in the non-dexa group in first 2 years since primary surgery. The cumulative incidence of PJI in TKA and THA was 0.78% (29 of 3695) and 0.48% (25 of 5157) respectively. Among 54 PJIs, 11 cases (21% of PJIs) of *S. aures* infection, 8 cases (15%) of *S. agalactiae*, 5 cases (9%) of *S. dysgalactiae*, 4 cases (8%) *S. epidermidis*, 9 cases (17%) of polymicrobial infection and 10 cases (19%) of culture negative infection. Considering the 10 cases of culture negative PJI, all those cases either sinus tract was present (6 of 10) or have positive result of histology (10 of 10) of the intraoperative samples. Among those patients 7 (70%) of them received antibiotics prior to sampling. In the remaining 3 (30%) cases without preoperative antibiotics, in addition to histology also the intraoperative synovial fluid cell count with differential were consistent with the infection. The detailed data on isolated bacteria are presented in Table 3. Within first 2 postoperative years in total 120 revisions were registered, from them 54 (45%) due to PJI and 66 (55%) due to aseptic reasons. Considering the aseptic revisions, there were 41 (62%) of THA and 25 (38%) of TKA. The detailed causes of aseptic revisions of THA and TKA are provided in Table 4.

**Table 2:** Univariate analysis of variable association with PJI and all causes of revision within 2 years since primary surgery.

n	Revision for PJI				Revision for all causes			
	No	Yes	OR (95% CI)	*P* value	No	Yes	OR (95% CI)	*P* value
	8851	54			8785	120		
Age (y)	69.1 (62.6-75.3)	70 (63-75.9)	1.01 (0.98-1.04)	.675	69.1 (62.6-75.4)	69.4 (62.7-77.3)	1.01 (0.99-1.03)	.506
Sex, male	3895 (44%)	34 (63%)	2.16 (1.26-3.83)	.006	3869 (44%)	60 (50%)	1.27 (0.89-1.82)	.193
BMI (kg/m^2^)	29.8 (26.6-33.5)	33.4 (30.3-37)	1.13 (1.08-1.18)	<.001	29.8 (26.6-33.5)	32.8 (28.7-35.1)	1.08 (1.05-1.11)	<.001
ASA								
I-II	5398 (61%)	22 (41%)	Ref.		5361 (61%)	59 (49%)	Ref.	
III-IV	3453 (39%)	32 (59%)	2.27 (1.33-3.97)	.003	3424 (39%)	61 (51%)	1.56 (1.19-2.14)	.002
Diabetes								
No	7924 (90%)	49 (91%)	Ref.		7866 (90%)	107 (89%)	Ref.	
Yes	927 (10%)	5 (9%)	0.87 (0.30-1.99)	.772	919 (10%)	13 (11%)	1.04 (0.58-1.86)	.895
Joint								
Knee	3694 (42%)	29 (54%)	Ref.		3669 (42%)	54 (45%)	Ref.	
Hip	5157 (58%)	25 (46%)	0.62 (0.36-1.06)	.078	5116 (58%)	66 (55%)	0.88 (0.61-1.26)	.476
Dexa								
No	7539 (85%)	48 (89%)	Ref.		7483 (85%)	104 (87%)	Ref.	
Yes	1312 (15%)	6 (11%)	0.72 (0.27-1.55)	.446	1302 (15%)	16 (13%)	0.88 (0.52-1.50)	.649

Ref., reference group.

**Table 3:** Microbes isolated among PJIs in our cohort within first 2 postoperative years.

Microbe	n (%)
*S. aureus*	11 (21)
*S. dysgalactiae*	5 (9)
*S. agalactiae*	8 (15)
*S. mitis*	1 (2)
*S. pyogenes*	1 (2)
*S. pneumoniae*	1 (2)
*E. faecalis*	1 (2)
*S. epidermidis*	4 (8)
*S. lugdunensis*	1 (2)
*S. canis*	1 (2)
*Pasteurella multocida*	1 (2)
Polymicrobial	9 (17)
Culture negative	10 (19)

**Table 4:** Reasons for revision of THAs and TKAs included in the cohort with corresponding frequencies at 2-year follow-up.

Reason for revision	THA	TKA	*P value*
	n = 66	n = 54	
Septic	25 (38%)	29 (54%)	.083
Aseptic	41 (62%)	25 (46%)	
Periprosthetic loosening	11 (17%)	6 (11%)	
Periprosthetic fracture	16 (24%)	5 (9%)	
Dislocation	8 (12%)	-	
Component malpositioning	3 (5%)	-	
Leg length discrepancy	1 (2%)	-	
Hematoma	2 (3%)	-	
Instability	-	7 (13%)	
Patellofemoral pain	-	6 (11%)	
Allergy to the implant	-	1 (2%)	

n, number of cases; %, percentage of the revisions in the subgroup.

In multiple logistic regression analysis, adjusted for sex, BMI, operated joint and ASA, dexa remained not significant risk factor for infection (OR 0.80 95% CI 0.3-1.73; *P* = .603) (Table 5). In the multivariate analysis, male sex and BMI were associated with increased risk for PJI (OR 2.52, 95% CI 1.45-4.51, *P* = .001) and (OR 1.12, 95% CI 1.07-1.17, *P* < .001) respectively. The increase of BMI for 1 kg/m^2^ corresponds to increased odds for PJI for 12% in a person of the same sex, ASA, operated joint and dexa administration. While ASA (III-IV vs I-II; OR 1.7, 95% CI 0.92-3.02, *P* = .067) was close to statistical significance.

**Table 5:** Results of multiple logistic regression.

	OR (95% CI)	*P* value
Sex, male	2.52 (1.45-4.51)	.001
BMI (kg/m^2^)	1.12 (1.07-1.17)	<.001
ASA		
I-II	Ref.	
III-IV	1.70 (0.92-3.02)	.067
Joint		
knee	Ref.	
hip	0.79 (0.45-1.37)	.398
Dexa		
no	Ref.	
yes	0.80 (0.30-1.73)	.603

Ref., reference group.

## Discussion

Well-established effects of perioperative use of corticosteroids include reduced postoperative nausea, pain, and enhanced recovery after THA and TKA [10]. However, there are still several concerns about the safety of perioperative corticosteroid administration regarding increased risk for PJI [3,4,15]. The exact dose and timing of dexamethasone administration varied extremely in the literature available. Similarly, the efficacy and preferred type of corticosteroid used as well as the type of administration are extremely varied [9,10,[16], [17], [18]]. In a recent study, Heckmann et al. examined the results of 1,322,025 primary elective total joint arthroplasties from over 1000 hospitals and showed no association of intraoperative dexamethasone use with increased risk of PJI within 90 days postoperatively [11]. We analyzed a single systemic intravenous administration of low-dose dexamethasone 0.15 mg/kg approximately 15 minutes before the start of the surgery in a single hospital with standard PJI diagnostic procedures in all patients undergoing total joint arthroplasty revision surgery with minimal 2-year follow-up and found no association with an increased risk of PJI in THA and TKA in patients with primary osteoarthritis.

In previous evaluations, power analysis indicated that at least 3500 patients would be required to adequately assess an increase in PJI [16]. Given the observational nature of this study, the post-hoc power was estimated at 0.45, suggesting a substantial risk (55%) of failing to detect a true difference if one exists. This highlights the need for caution when interpreting non-significant results, as the study may have been underpowered to identify modest but potentially clinically relevant associations. Although still underpowered, this study is the largest single hospital cohort study where PJI has been studied as the primary outcome after perioperative intravenous administration of single-dose dexamethasone in THA and TKA done only in patients with primary osteoarthritis. Our data show that perioperative administration of dexamethasone was not associated with a higher risk for PJI (OR 0.80 95% CI 0.30-1.73; *P* = .603) within the first 2 postoperative years. Findings support previous reports showing no difference in PJI incidence after TKA and THA when dexamethasone has been used perioperatively. Most to date available studies report the rate of PJI within 1 year after primary surgery; however, it has been reported that most of low-grade PJIs clinically present within first 2 postoperative years [19]. Richardson et al. [2] in their study with a total of 6294 and 557 patients in the dexamethasone group with different preoperative diagnoses reported observed PJI rate at 1 year in the dexamethasone group to be 0.8% compared to 0.9% in the control group, at 6-year follow-up the observed PJI rate increased to 1.3% and 1.2% respectively and remain statistically not significant (*P* = .80). Similarly, Vourinen et al. [7] in their study included a total of 18,872 procedures (THA, TKA, and revisions) with 2922 patients in the dexamethasone group and the observed rate of PJI in the dexamethasone group was 1.1% compared to 1.0% in the control group which was not statistically significant *P* = .77 at mean follow-up 4.9 years (range 1.0-9.7). However, the effect of preoperative diagnosis was not analyzed. Recent systemic reviews with meta-analysis report that to date evidence indicates no increased risk of PJI. Freeley et al. in their meta-analysis included 29 studies using either a single low or high dose of corticosteroid in the perioperative period compared with the control group without dexamethasone use and they noted no difference in PJI risk either for THA (*P* = .59) or for TKA (*P* = .2) [9]. Lex et al. in systemic review with meta-analysis analyzed 17 different randomized controlled trials using dexamethasone in the perioperative period with 1144 patients included in the intervention group and observed no difference in occurrence of deep as well as superficial infections in group with administered dexamethasone [20].

However, we are aware of the limitations of our study, as follows: the study is still underpowered to fully evaluate a rare event, such as PJI. In previous evaluations, it has been calculated with the power analysis that at least 3500 patients should be included to fully evaluate the increase in PJI [16]. Given the observational nature of this study, the post-hoc power was estimated at 0.45, which suggests a substantial risk (55%) of failing to detect a true difference if one were present. This underscores the need for caution when interpreting non-significant results, as the study may not have been sufficiently powered to identify modest but potentially meaningful associations. Furthermore, this study is a retrospective analysis of prospectively collected data and patients were not randomized. The use of dexamethasone was decided upon by the anesthesiologist individually. Smoking as a possible risk factor for PJI was not analyzed due to a lack of reliable data. This study analyzed only a single low-dose intravenous administration of dexamethasone while the types of administration protocols (suitable timing and dose of dexamethasone) used to achieve the highest efficacy with the lowest complication rate extremely varied in the literature [2,9,16,21]. In this respect, the results of our study cannot be fully generalized.

## Conclusions

Our study is the first single-dose, single-hospital study with the standardized PJI diagnostic procedures used at all joint arthroplasty revision surgeries which shows that intraoperative administration of intravenous dexamethasone has no association with an increased risk of early or delayed PJI in THA and TKA in patients with primary osteoarthritis. The present data provide evidence in support of intraoperative administration of a single dose of dexamethasone in primary THA and TKA.

## CRediT authorship contribution statement

**Samo Roškar:** Writing – review & editing, Writing – original draft, Visualization, Validation, Software, Methodology, Investigation, Formal analysis, Conceptualization. **Vesna Levašič:** Writing – review & editing, Writing – original draft, Visualization, Validation, Supervision, Software, Resources, Project administration, Methodology, Investigation, Formal analysis, Data curation, Conceptualization. **Mateja Blas:** Writing – review & editing, Writing – original draft, Visualization, Validation, Software, Methodology, Investigation, Formal analysis, Data curation, Conceptualization. **Simon Kovač:** Writing – review & editing, Writing – original draft, Visualization, Validation, Supervision, Software, Resources, Methodology, Investigation, Funding acquisition, Formal analysis, Data curation, Conceptualization.

## Appendix ASupplementary data

Conflict of Interest Statement for Blas

Conflict of Interest Statement for Kovač

Conflict of Interest Statement for Levašič

Conflict of Interest Statement for Roškar
